# One-year results of treat-and-extend regimen with intravitreal brolucizumab for treatment-naïve neovascular age-related macular degeneration with type 1 macular neovascularization

**DOI:** 10.1038/s41598-022-10578-1

**Published:** 2022-05-17

**Authors:** Hidetaka Matsumoto, Junki Hoshino, Ryo Mukai, Kosuke Nakamura, Hideo Akiyama

**Affiliations:** Department of Ophthalmology, Gunma University Graduate School of Medicine, 3-39-15 Showa-machi, Maebashi, Gunma 371-8511 Japan

**Keywords:** Diseases, Eye diseases, Retinal diseases

## Abstract

We evaluated 1-year outcomes of loading phase treatment followed by maintenance treatment using a treat-and-extend (TAE) regimen with intravitreal brolucizumab for neovascular age-related macular degeneration (nAMD) associated with type 1 macular neovascularization (MNV). We analyzed 68 eyes of 65 consecutive patients with treatment-naïve nAMD associated with type 1 MNV. Forty-five eyes (66.2%) completed the 1-year treatment with intravitreal brolucizumab. In those cases, best-corrected visual acuity (BCVA) showed significant improvement, while there were significant reductions in foveal thickness and central choroidal thickness, after the initial brolucizumab injection, which were maintained until the last visit. The average total number of injections over 1 year was 6.4 ± 0.6. The average intended injection interval at the last visit was 14.0 ± 2.9 weeks. Moreover, 17of 23 eyes (73.9%) with polypoidal lesions showed complete regression of these lesions after the loading phase treatment. Although intraocular inflammation (IOI) was observed in 15 of 68 eyes (22.1%) within 1 year, amelioration in response to combination therapy with topical and subtenon injection of steroids, without visual decline, was obtained. These results indicate that loading phase treatment followed by the TAE regimen with intravitreal brolucizumab might improve BCVA and ameliorate exudative changes in eyes with treatment-naïve nAMD associated with type 1 MNV. Moreover, intravitreal brolucizumab can potentially reduce the treatment burden of nAMD. Prompt steroid therapy might be efficacious for ameliorating brolucizumab-related IOI without visual decline.

## Introduction

Various approaches have been used to treat neovascular age-related macular degeneration (nAMD). Currently, intravitreal injection of an anti-vascular endothelial growth factor (VEGF) agent is the first-line treatment, which can improve and maintain visual acuity in patients with nAMD^1^. Intravitreal anti-VEGF agent administration is divided into loading and maintenance phases. The loading phase usually consists of 3 monthly intravitreal injections, while the maintenance phase is basically divided into 3 regimens: fixed, pro re nata (PRN), and treat-and-extend (TAE)^1^. Systematic reviews have demonstrated the efficacy of the TAE regimen to be superior to that of the PRN regimen^2^. Moreover, the TAE regimen has proven to be as effective as the fixed regimen with fewer injections^3^. Therefore, TAE is the most widely recommended regimen for the treatment of nAMD in clinical practice^1,4^.

Several anti-VEGF agents, including ranibizumab and aflibercept, have been used to treat nAMD. Brolucizumab, a novel anti-VEGF agent for nAMD, was launched in 2019 in the US and has been available in Japan since 2020. Brolucizumab is a humanized single-chain antibody fragment with a molecular mass of 26 kDa, characterized by its small molecular mass and high solubility compared to other anti-VEGF agents^5^. The HAWK and HARRIER phase 3 clinical trials revealed that intravitreal brolucizumab 6 mg was non-inferior to intravitreal aflibercept 2 mg for improving and maintaining visual acuity in eyes with nAMD^6,7^. Moreover, intravitreal brolucizumab 6 mg was more effective than intravitreal aflibercept 2 mg for controlling not only intraretinal and subretinal fluid but also sub-retinal pigment epithelium (RPE) fluid^6,7^. Based on these findings, we have been using brolucizumab especially for nAMD with type 1 macular neovascularization (MNV). In 2021, we reported the results of loading phase treatment with intravitreal brolucizumab for 42 eyes with treatment-naïve nAMD associated with type 1 MNV^8^. In 36 eyes (85.7%) that completed 3 monthly injections of brolucizumab, best-corrected visual acuity (BCVA) showed significant improvement, while foveal thickness and central choroidal thickness (CCT) were significantly reduced during the loading phase treatment. In addition, polypoidal lesions showed complete regression in 15 of the 19 eyes (78.9%) with these lesions. Although intraocular inflammation (IOI) was observed in 8 of 42 eyes (19.0%), prompt steroid therapy ameliorated the IOI without visual decline from the baseline after 3 months.

In the present study, we evaluated 1-year outcomes of loading phase treatment followed by maintenance treatment using a TAE regimen with intravitreal brolucizumab for treatment-naïve nAMD associated with type 1 MNV.

## Results

The subjects were 68 eyes of 65 patients (56 eyes of 53 men; 12 eyes of 12 women, average age: 75.4 ± 8.9 years) with treatment-naïve nAMD associated with type 1 MNV. Forty-five eyes (66.2%) of 42 patients (38 eyes of 35 men; 7 eyes of 7 women, average age: 73.1 ± 8.2 years) completed 1 year of treatment with brolucizumab, 15 eyes (22.1%) of 15 patients (10 men and 5 women, average age: 80.9 ± 6.4 years) developed IOI and stopped brolucizumab treatment, and 8 eyes (11.8%) of 8 patients (8 men, average age: 77.8 ± 10.9 years) dropped out within 1 year after starting the treatment.

**Figure 1:**
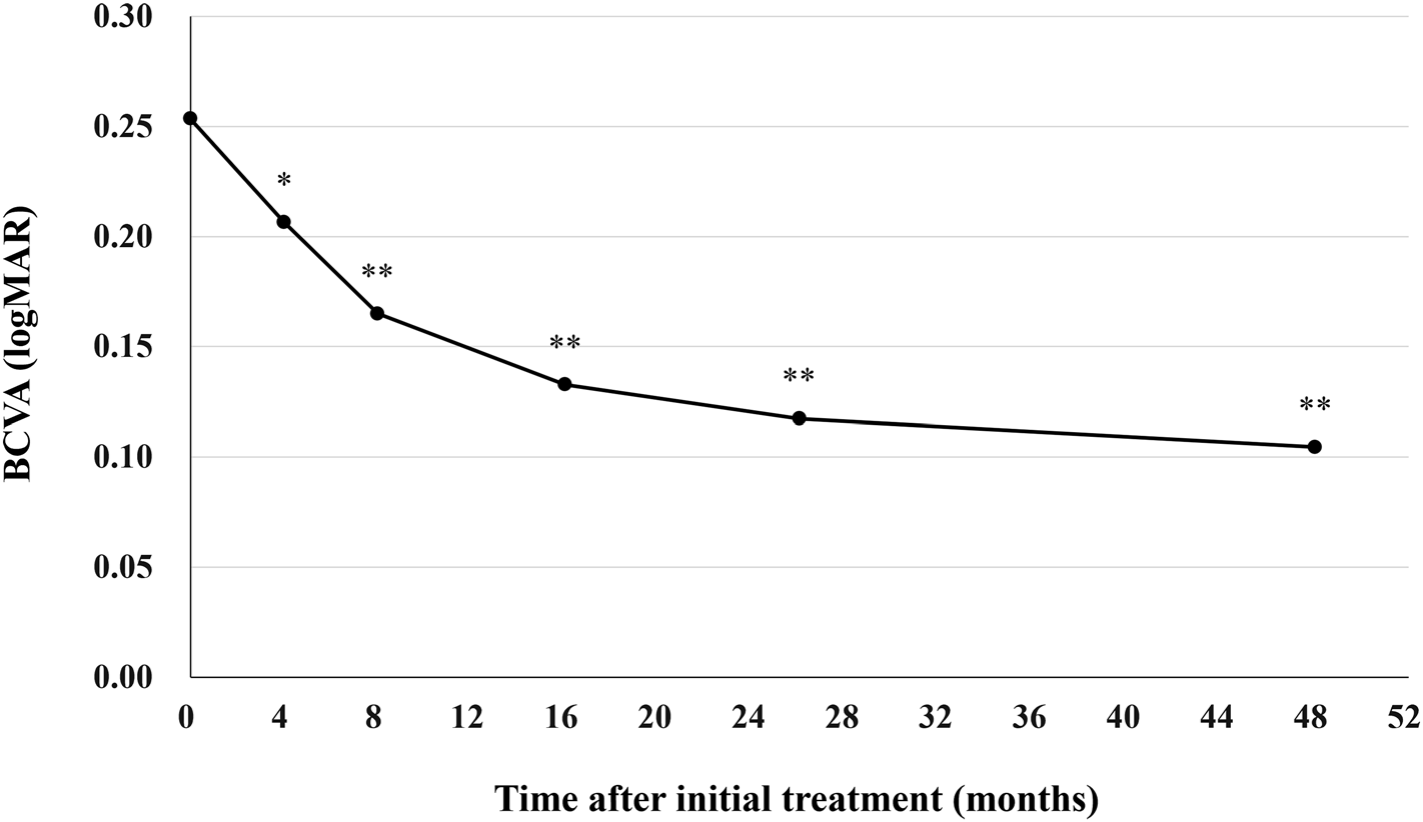
Change of average best-corrected visual acuity (BCVA) in 45 eyes with neovascular age-related macular degeneration associated with type 1 macular neovascularization treated with 3 monthly intravitreal injections of brolucizumab followed by a treat-and-extend regimen with intravitreal brolucizumab. BCVA showed significant improvement after the first injection of brolucizumab (*P < 0.05, **P < 0.01). Data are expressed as averages.

**Figure 2:**
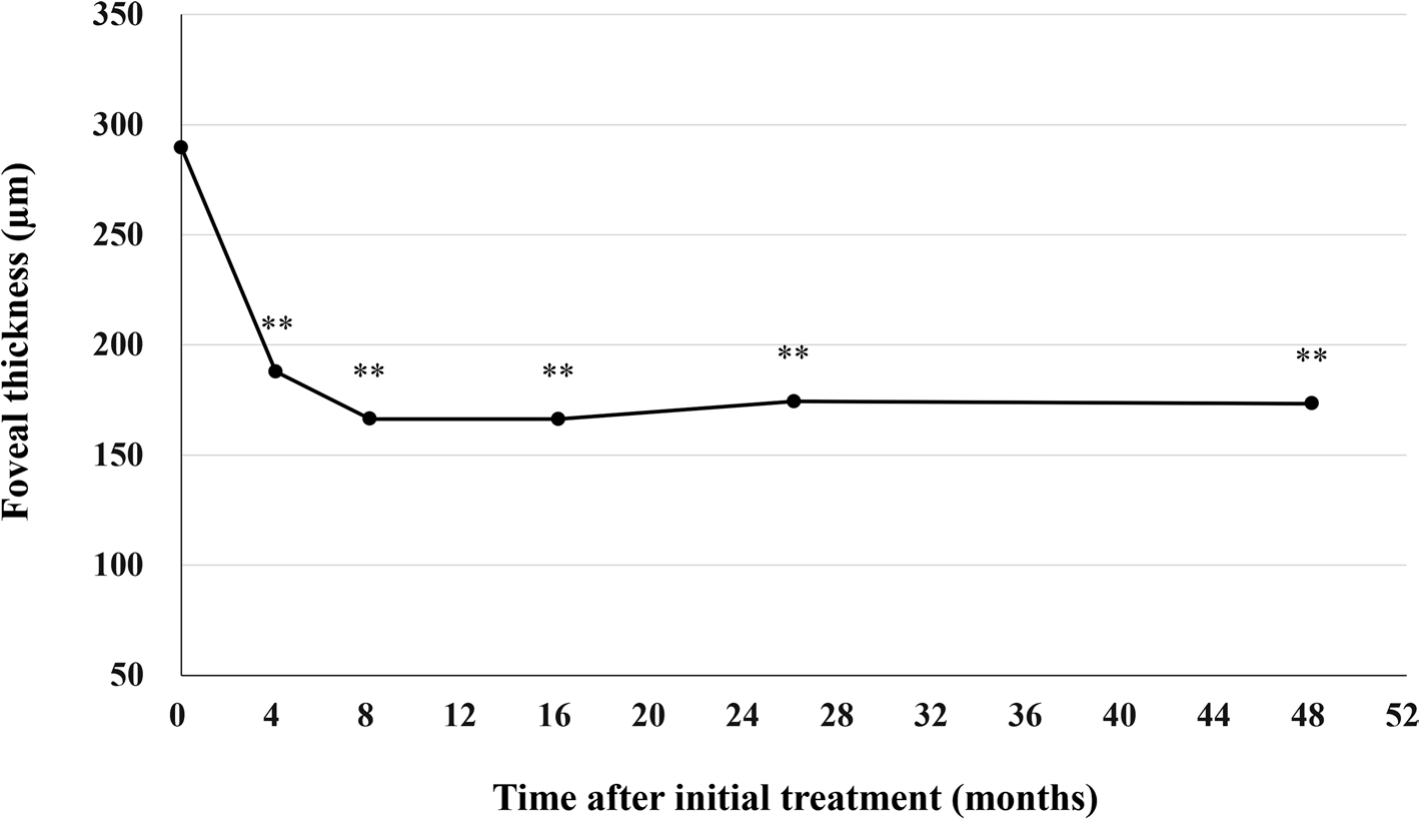
Change of average foveal thickness in 45 eyes with neovascular age-related macular degeneration associated with type 1 macular neovascularization treated with 3 monthly intravitreal injections of brolucizumab followed by a treat-and-extend regimen with intravitreal brolucizumab. Foveal thickness showed significant reduction after the first injection of brolucizumab (**P < 0.01). Data are expressed as averages.

**Figure 3:**
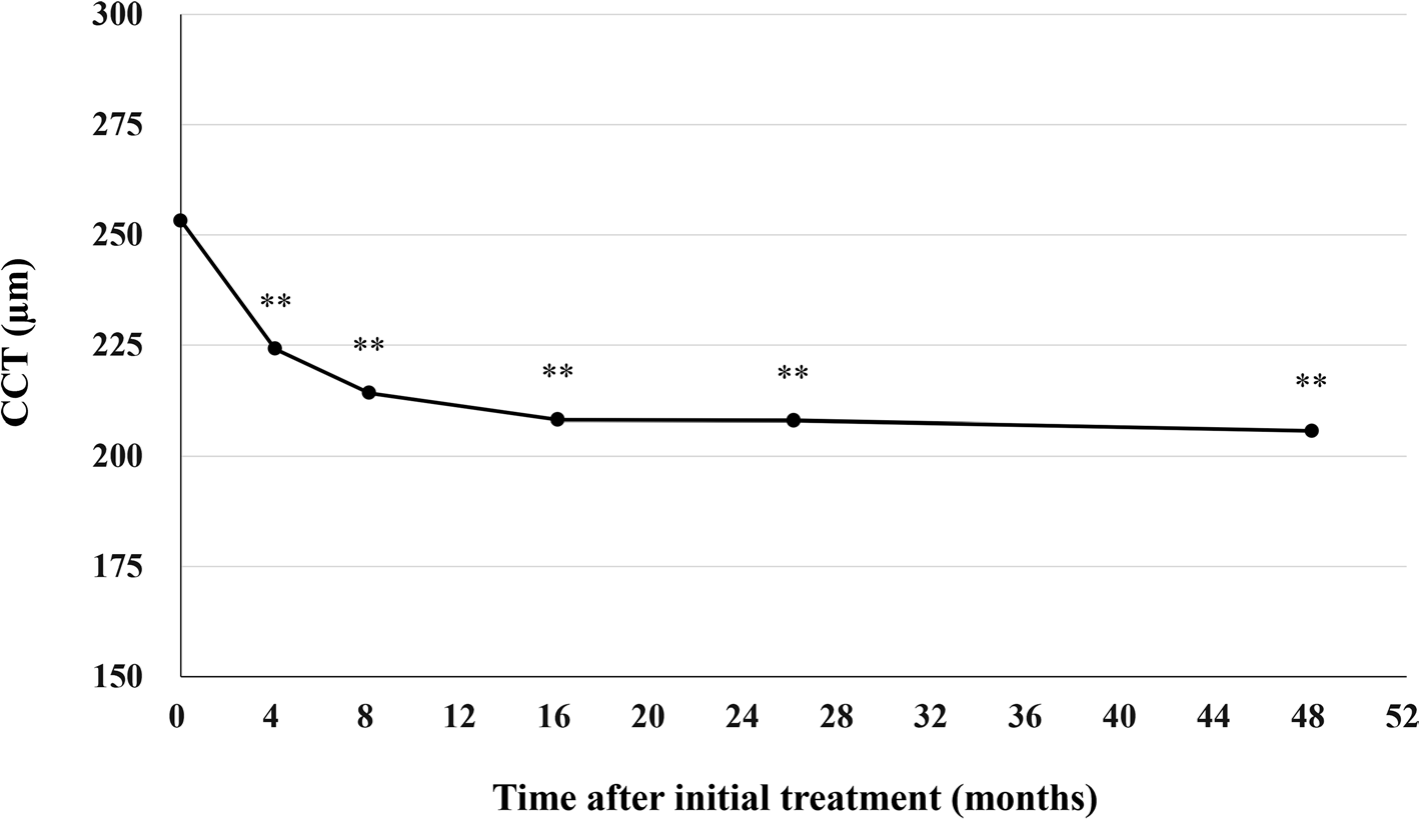
Change of average central choroidal thickness (CCT) in 45 eyes with neovascular age-related macular degeneration associated with type 1 macular neovascularization treated with 3 monthly intravitreal injections of brolucizumab followed by a treat-and-extend regimen with intravitreal brolucizumab. CCT showed significant reduction after the first injection of brolucizumab (**P < 0.01). Data are expressed as averages.

**Figure 4:**
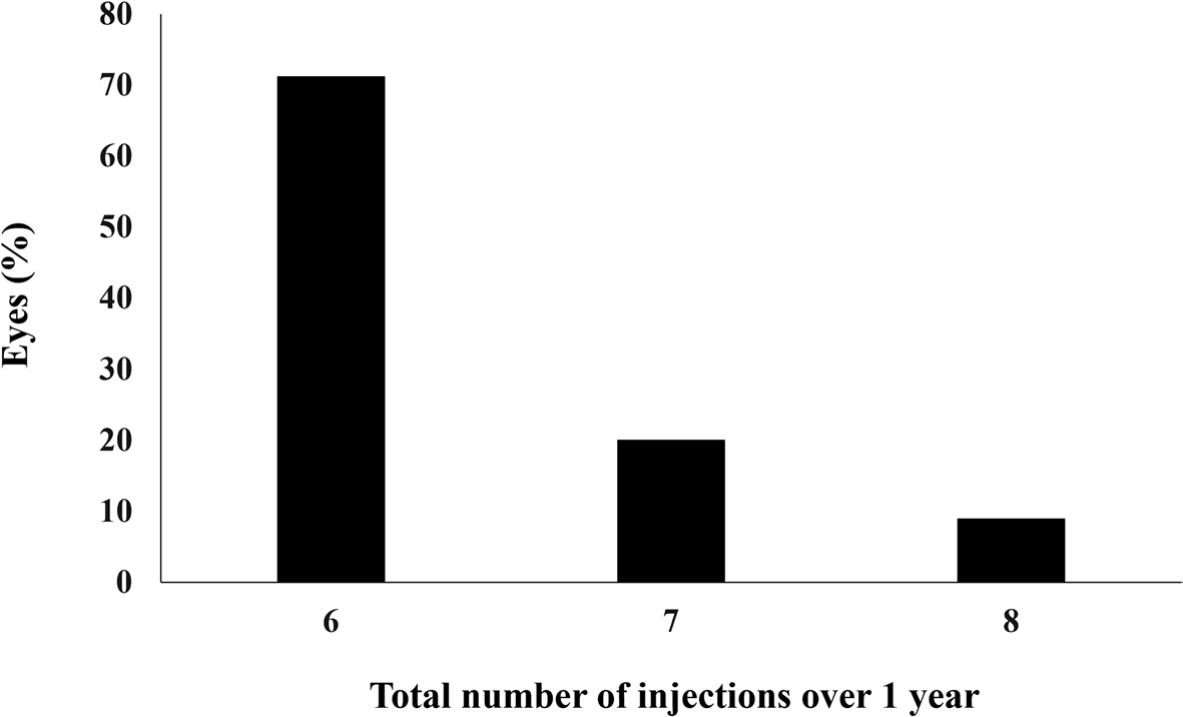
Total number of injections over the 1-year study period in 45 eyes with neovascular age-related macular degeneration associated with type 1 macular neovascularization treated with 3 monthly intravitreal injections of brolucizumab followed by a treat-and-extend regimen with intravitreal brolucizumab.

**Figure 5:**
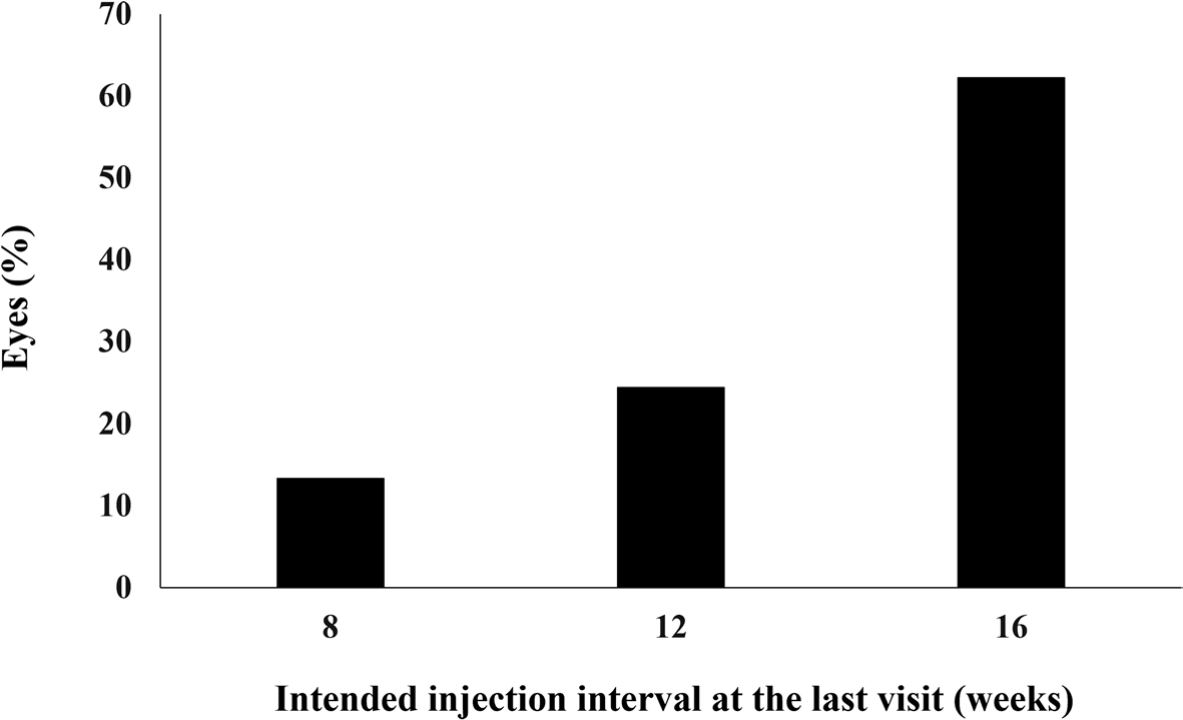
Intended injection interval at the last visit up to week 52 in 45 eyes with neovascular age-related macular degeneration associated with type 1 macular neovascularization treated with 3 monthly intravitreal injections of brolucizumab followed by a treat-and-extend regimen with intravitreal brolucizumab.

**Figure 6:**
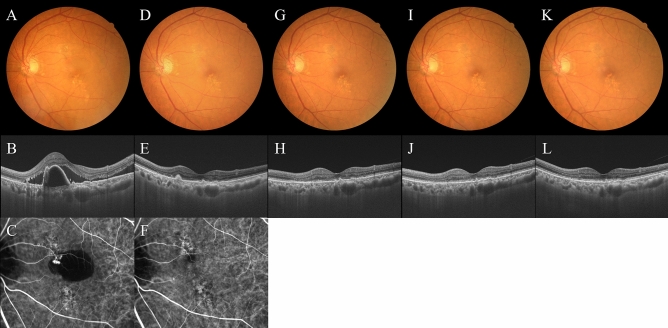
Images of the left eye of a 76-year-old man with polypoidal choroidal vasculopathy. The results of loading phase treatment with brolucizumab for this eye were shown in our prior report^8^. At baseline, best-corrected visual acuity (BCVA) was 0.22 logarithm of the minimum angle of resolution (logMAR) units. (**A**) Color fundus photograph shows retinal pigment epithelium (RPE) degeneration and detachment accompanied by serous retinal detachment (SRD) at the macular area. (**B**) 12 mm B-mode optical coherence tomography (OCT) image through the fovea and polypoidal lesion shows sharply peaked RPE detachment due to the polypoidal lesion, which is accompanied by serous RPE detachment and SRD. Moreover, choroidal thickening associated with dilatation of outer choroidal vessels is seen in the area of the double layer sign reflecting a branching neovascular network. The foveal thickness and CCT are 388 μm and 360 μm, respectively. (**C**) Indocyanine green angiography (ICGA) shows polypoidal lesions in the RPE detachment and a branching neovascular network. At week 12, 4 weeks after the third injection of brolucizumab: BCVA of the left eye is -0.08 logMAR units. (**D**) Color fundus photograph shows RPE degeneration at the macular area. (**E**) 12 mm B-mode OCT image shows neither serous RPE detachment nor SRD. The foveal thickness and CCT are 148 μm and 303 μm, respectively. (**F**) ICGA shows no polypoidal lesions. At week 16: BCVA of the left eye is -0.08 logMAR units. (**G**, **H**) Color fundus photograph and 12 mm B-mode OCT image shows no recurrence of exudative changes. The foveal thickness and CCT are 165 μm and 313 μm, respectively. The fourth injection of brolucizumab was administered with an interval of 8 weeks. At week 28: BCVA of the left eye is -0.08 logMAR units. (I) (J) Color fundus photograph and 12 mm B-mode OCT image shows no recurrence of exudative changes. The foveal thickness and CCT are 146 μm and 313 μm, respectively. The fifth injection of brolucizumab was administered with an interval of 12 weeks. At week 44: BCVA of the left eye is − 0.08 logMAR units. (**K**, **L**) Color fundus photograph and 12 mm B-mode OCT image show no recurrence of exudative changes. The foveal thickness and CCT are 162 μm and 312 μm, respectively. The sixth injection of brolucizumab was administered with an interval of 16 weeks. The intended interval for the next injection of brolucizumab is also 16 weeks. No adverse events such as intraocular inflammation were observed during the 1-year treatment period.

All patients who completed the 1 year of brolucizumab treatment visited on the scheduled days. In these cases, BCVA at baseline, week 4, 8, 16, either 24 or 28, and finally 44, 48, or 52 were 0.25 ± 0.30, 0.21 ± 0.28 (*P* < 0.05), 0.16 ± 0.27 (*P* < 0.01), 0.13 ± 0.28 (*P* < 0.01), 0.12 ± 0.25 (*P* < 0.01), and 0.10 ± 0.26 (*P* < 0.01), respectively. BCVA showed significant improvement after the first brolucizumab injection (Fig. 1). Foveal thickness at baseline, week 4, 8, 16, either 24 or 28, and finally 44, 48, or 52 were 290 ± 105, 188 ± 68 (*P* < 0.01), 166 ± 52 (*P* < 0.01), 166 ± 49 (*P* < 0.01), 174 ± 58 (*P* < 0.01), and 173 ± 56 (*P* < 0.01), respectively. Foveal thickness was significantly decreased after the first intravitreal brolucizumab administration (Fig. 2). CCT at baseline, week 4, 8, 16, either 24 or 28, and finally 44, 48, or 52 were 253 ± 91, 224 ± 86 (*P* < 0.01), 214 ± 84 (*P* < 0.01), 208 ± 81 (*P* < 0.01), 208 ± 79 (*P* < 0.01), and 206 ± 80 (*P* < 0.01), respectively. CCT was significantly decreased after the first brolucizumab injection (Fig. 3). The total number of injections over the 1-year study period was 6 in 32 eyes (71.1%), 7 in 9 eyes (20.0%), and 8 in 4 eyes (8.9%), with the number of injections averaging 6.4 ± 0.6 (Fig. 4). The intended injection interval at the last visit was 8 weeks in 6 eyes (13.3%), 12 weeks in 11 eyes (24.4%), and 16 weeks in 28 eyes (62.2%), with an average injection interval of 14.0 ± 2.9 weeks (Fig. 5). During the maintenance phase, 26 eyes (57.8%) showed no recurrence of exudative changes and the injection interval was successfully extended, while only 1 eye (2.2%) stayed at the 8-week interval due to persistent exudative changes. A representative case is shown in Fig. 6.

Dry macula was achieved in 39 eyes (86.7%) at week 16. The total number of injections for the 1-year study period was significantly lower in the cases that achieved dry macula at week 16 than in the other cases (6.2 ± 0.5 vs 7.3 ± 0.7, *P* < 0.01). Moreover, the intended injection interval at the last visit was significantly longer in those that achieved dry macula at week 16 than in the other cases (14.3 ± 2.7 vs 12.0 ± 3.0, *P* < 0.05) (Table 1).

**Table 1:** Comparison between eyes with and without dry macula at week 16.

	Dry macula (+)	Dry macula (−)	*P* value
Number of eyes	39 (86.7%)	6 (13.3%)	
Number of injections for 1 year	6.2 ± 0.5	7.3 ± 0.7	< 0.01
Intended injection interval at the last visit (weeks)	14.3 ± 2.7	12.0 ± 3.0	< 0.05

Twenty-three eyes (51.1%) in this study showed type 1 MNV with polypoidal lesions. Indocyanine green angiography (ICGA) after the loading phase treatment revealed complete regression of the polypoidal lesions in 17 of these 23 (73.9%) eyes. The total number of injections for the 1-year study period was significantly lower in cases showing complete regression of polypoidal lesions after the loading phase than in the other patients (6.1 ± 0.2 vs. 6.8 ± 0.9, *P* < 0.05). Furthermore, the intended injection interval at the last visit was significantly longer in those showing complete regression of polypoidal lesions after the loading phase than in the other patients (15.5 ± 1.3 vs. 12.7 ± 3.6, *P* < 0.05) (Table 2).

**Table 2:** Comparison between eyes with and without complete regression of polypoidal lesions after the loading phase treatment.

	Complete regression of polypoidal lesions (+)	Complete regression of polypoidal lesions (−)	*P* value
Number of eyes	17 (73.9%)	6 (26.1%)	
Number of injections for 1 year	6.1 ± 0.2	6.8 ± 0.9	< 0.05
Intended injection interval at the last visit (weeks)	15.5 ± 1.3	12.7 ± 3.6	< 0.05

Forms of brolucizumab-related IOI, such as iritis, vitritis, retinal vasculitis, retinal vascular occlusion, and papillitis, were observed in 6, 10, 10, 2, and 1 eye, respectively. Fourteen of the 15 eyes (93.3%) with IOI developed this complication within 3 months after the initial treatment. One eye underwent vitrectomy due to submacular hematoma and vitreous hemorrhage without subtenon injection of triamcinolone acetonide. The remaining 14 eyes were promptly managed with combination therapy using 0.1% betamethasone eye drops and a subtenon injection of 30 mg triamcinolone acetonide. After amelioration of IOI, 13 eyes continued the anti-VEGF therapy with aflibercept using the PRN or the TAE regimen and 1 eye received a single administration of photodynamic therapy. BCVA in the 15 eyes with IOI improved significantly from 0.34 ± 0.40 at baseline to 0.20 ± 0.42 at week 47 ± 4, the average timepoint of the last visit (*P* < 0.01). The final BCVA was better than baseline in 14 eyes and was the same as that at baseline in the 1 eye that developed submacular hematoma and vitreous hemorrhage. There was no significant difference in BCVA improvement during the 1-year study period between eyes with and without brolucizumab-related IOI (P = 0.82).

Eight eyes of eight patients dropping out within 1 year after the initial treatment had all completed the loading phase treatment. In these cases, BCVA showed significant improvement from 0.52 ± 0.38 at baseline to 0.37 ± 0.37 at week 12 (*P* < 0.05).

## Discussion

We investigated 1-year results of the TAE regimen with intravitreal brolucizumab for 68 eyes with treatment-naïve neovascular AMD associated with type 1 MNV. Forty-five eyes (66.2%) completed the 1-year treatment with intravitreal brolucizumab. In those cases, BCVA showed significant improvement, while foveal thickness and CCT were significantly reduced after the initial injection of brolucizumab and these reductions persisted until the last visit. The average total number of injections over the 1-year study period was 6.4 ± 0.6. The average intended injection interval at the last visit was 14.0 ± 2.9 weeks. During the maintenance phase, 57.8% of the eyes showed no recurrence of exudative changes. Moreover, 73.9% of the eyes with polypoidal lesions showed complete regression of these lesions after the loading phase treatment. Although brolucizumab-related IOI was observed in 15 of 68 eyes (22.1%) within 1 year, this complication showed amelioration, without visual decline, in response to combination therapy with topical and subtenon injection of steroids.

ALTAIR was a randomized, open-label, phase 4 study on the efficacy and safety of intravitreal aflibercept with two different TAE regimens (2- and 4-week adjustments) in Japanese patients with nAMD, which allowed a minimum interval of 8 weeks and a maximum interval of 16 weeks^9^. In the 4-week adjustment group consisting of 123 eyes, the proportion of patients without fluid was 62.6% at week 16. The intended injection interval at the last visit up to week 52 was an average of 11.8 ± 3.7 weeks, with 8 weeks for 39.8% and 16 weeks for 40.7% of patients. The proportion of patients who stayed at an 8-week injection interval was 22.0%. In the present study, dry macula was achieved at week 16 in 86.7% of eyes completing 1 year of brolucizumab treatment. The intended injection interval at the last visit was an average of 14.0 ± 2.9 weeks, with 8 weeks for 13.3% and 16 weeks for 62.2% of eyes. The proportion of patients who stayed at an 8-week injection interval due to persistent exudative changes was 2.2%. Although our current study results cannot be directly compared with those of the ALTAIR study because of some differences in the inclusion criteria and among the TAE regimens, brolucizumab appears to be more effective than aflibercept in ameliorating exudative changes, thereby reducing the number of injections in the TAE regimen.

In this study, the total number of injections was significantly lower and the intended injection interval at the last visit was significantly longer in the cases achieving dry macula at week 16 than in the other cases. Similar results were obtained in the ALTAIR study^10^, indicating that dry macula at week 16 may be a useful parameter for predicting subsequent treatment outcomes with the TAE regimen. Therefore, the high rate of dry macula (86.7%) at week 16 with brolucizumab treatment raises the possibility of reducing the burden of nAMD treatment.

The rate of polypoidal choroidal vasculopathy (PCV) in nAMD is reportedly higher in Asian than in Caucasian patients^11^. We reported 2-year results of a TAE regimen with aflibercept for PCV in 2017 and found that patients with complete regression of polypoidal lesions after the loading phase treatment required significantly fewer injections over the 2-year treatment period than those with residual polypoidal lesions^12^. In the present study, the number of injections over the 1-year study period was significantly lower and the final injection interval was significantly longer in cases with complete regression of polypoidal lesions after the loading phase treatment than in those with residual polypoidal lesions. Thus, regression of polypoidal lesions might be one of the goals of PCV treatment aimed at achieving favorable outcomes and reducing the treatment burden. The regression rate of polypoidal lesions after the loading phase treatment was 73.9% in the present study, which is similar to the rates obtained in previous studies using brolucizumab^8,13^. In contrast, the regression rates of polypoidal lesions after the loading phase treatment were reported to be approximately 30% with ranibizumab and approximately 50% with aflibercept^12,14–16^. Therefore, compared with ranibizumab and aflibercept, brolucizumab achieves a higher regression rate of polypoidal lesions and may thus be a useful anti-VEGF agent for treating PCV.

A post hoc review of the HAWK and HARRIER studies found that 50 of 1088 eyes (4.6%) treated with brolucizumab developed IOI, of which 48% developed IOI within 3 months and 74% within 6 months after the first administration of this agent^17^. In our study, IOI occurred in 15 of 68 eyes (22.1%), 14 (93.3%) of which developed IOI within the first 3 months of starting treatment. Compared with the HAWK and HARRIER studies, the frequency of IOI was obviously higher, and the IOI onset was apparently earlier in this study. Other evaluations of real-world brolucizumab data in Japan also demonstrated a higher incidence of IOI than in the HAWK and HARRIER studies^8,13,18,19^. These results suggest that racial differences may play a role in the different responses to brolucizumab. The mechanism of IOI has not as yet been fully elucidated, although the involvement of anti-drug antibodies has been suggested as a possible cause of IOI^20^. However, as noted in the expert opinion on IOI management, it should be detected early and treated with intensive steroid therapy^21^. In our present study, prompt combination therapy with topical and subtenon injection of steroids successfully ameliorated IOI. Furthermore, after resolution of the IOI, additional treatments such as intravitreal aflibercept and PDT resulted in significant improvement of final BCVA as compared to the baseline. Moreover, there was no significant difference in BCVA improvement between eyes with and without IOI.

Limitations of our study include the retrospective single-center design, the small number of patients and the lack of controls. All subjects were Japanese, and the results may therefore not be generalizable to nAMD in Caucasians and other racial or ethnic groups. Moreover, this study assessed only the 1-year results of intravitreal brolucizumab therapy for nAMD with type 1 MNV including PCV. Thus, long-term outcomes and the efficacy of intravitreal brolucizumab for nAMD with other MNV subtypes still need to be evaluated.

In conclusion, loading phase treatment followed by a TAE regimen with intravitreal brolucizumab was effective for improving visual acuity and ameliorating exudative changes for 1 year in eyes with treatment-naïve nAMD associated with type 1 MNV. Moreover, relatively few visits and injections were required for this treatment. Prompt steroid therapy was efficacious for managing brolucizumab-related IOI and thereby preventing visual decline.

## Methods

We obtained approval from the Institutional Review Board of Gunma University Hospital and adhered to the guidelines of the Declaration of Helsinki in performing this study. Informed consent was obtained from all individual participants included in the study. We retrospectively studied 68 eyes of 65 consecutive patients with previously untreated nAMD associated with type 1 MNV. During the period from June 2020 through January 2021, the patients started to receive 3 monthly intravitreal injections of brolucizumab as a loading phase followed by a TAE regimen with intravitreal brolucizumab as a maintenance phase at Gunma University Hospital. We included in this study all eyes evaluated in our previous investigation of the outcomes of loading phase treatment with intravitreal brolucizumab for type 1 MNV secondary to nAMD^8^.

Before starting the treatment with intravitreal brolucizumab, all patients underwent complete ophthalmological examinations, including slit-lamp biomicroscopy with a noncontact fundus lens (SuperField lens; Volk Optical Inc, Mentor, OH), color fundus photography (Canon CX-1; Canon, Tokyo, Japan), ultra-widefield color fundus imaging (Optos 200Tx, Optos, Dunfermline, UK), fluorescein angiography (FA) and ICGA (Spectralis HRA + OCT; Heidelberg Engineering, Heidelberg, Germany), as well as swept-source OCT (DRI OCT-1 Triton; Topcon Corp, Tokyo, Japan, and PLEX Elite 9000; Carl Zeiss Meditec, Dublin, CA, USA). In the OCT examination, we obtained B-mode images of the horizontal and vertical line scans (12 mm) through the fovea employing the DRI OCT-1 Triton. Then, we performed OCT angiography (OCTA) volume scanning, i.e., 300 × 300 pixels in the 3 × 3 mm area demonstrated by the PLEX Elite 9000. The OCTA thus performed was based on an optical microangiography algorithm. The diagnostic criteria for nAMD were based on a previous report of nAMD nomenclature^22^. We diagnosed nAMD with type 1 MNV, if MNV was detected beneath the RPE by the aforementioned multimodal imaging. The presence of polypoidal lesions was evaluated on ICGA and B-mode OCT images, i.e., polyp-like choroidal vessel dilation on ICGA and sharply peaked RPE detachment on B-mode OCT^23^.

All eyes were treated with only intravitreal brolucizumab injection (6 mg/0.05 mL). In the loading phase, patients receive 3 monthly injections of brolucizumab. All patients again underwent FA and ICGA at week 12, 4 weeks after the third brolucizumab injection. In the maintenance phase, the interval of injections is extended by 4 weeks if there are no exudative changes, whereas the interval is shortened by 4 weeks in the event of any exudative change being seen. We set the treatment interval at a minimum of 8 weeks and a maximum of 16 weeks in this study. When non-infectious IOI developed, brolucizumab therapy was discontinued and 0.1% betamethasone eye drops (4–6 times/day) as well as posterior subtenon injection of triamcinolone acetonide (30 mg/0.5 mL) were basically administered.

At every visit, we conducted BCVA, slit-lamp biomicroscopy with a noncontact fundus lens, color fundus photography, ultra-widefield color fundus imaging, and swept-source OCT examinations. BCVA was determined with manifest refraction and recorded as decimal values and converted to the logarithm of the minimal angle of resolution (logMAR) units. Foveal thickness and CCT were measured on B-scan OCT images employing the computer-based caliper measurement tool in the OCT system. Foveal thickness was, by definition, the distance between the internal limiting membrane and the RPE surface at the fovea. Foveal thickness included any intraretinal and subretinal fluid. CCT was defined as the distance between Bruch’s membrane and the margin of the choroid and sclera under the fovea. Dry macula was defined as the macula without intraretinal, subretinal, and sub-RPE fluid accompanied by either no or diminishing hemorrhage.

For statistical analyses, the Wilcoxon signed-rank test was employed for comparison of the differences between BCVA, foveal thickness and CCT at baseline versus other timepoints. Unpaired values of BCVA, number of injections, and injection interval were compared using the Mann–Whitney *U* test. The data analyses were performed using Excel (Microsoft, Redmond, WA, USA) with add-in software Statcel4^24^. A *P* < 0.05 was considered to indicate a statistically significant difference. All data are presented as the average ± standard deviation.

## Data Availability

The datasets used and/or analyzed during the current study are available from the corresponding author on reasonable request.

## References

[1] Yamashiro K, Oishi A, Hata M, Takahashi A, Tsujikawa A (2021). Visual acuity outcomes of anti-VEGF treatment for neovascular age-related macular degeneration in clinical trials. Jpn J. Ophthalmol..

[2] Chin-Yee D, Eck T, Fowler S, Hardi A, Apte RS (2016). A systematic review of as needed versus treat and extend ranibizumab or bevacizumab treatment regimens for neovascular age-related macular degeneration. Br. J. Ophthalmol..

[3] Fallico M (2021). Treat and extend versus fixed regimen in neovascular age related macular degeneration: A systematic review and meta-analysis. Eur. J. Ophthalmol..

[4] Gale RP (2019). Action on neovascular age-related macular degeneration (nAMD): Recommendations for management and service provision in the UK hospital eye service. Eye (Lond.).

[5] Nguyen QD (2020). Brolucizumab: Evolution through preclinical and clinical studies and the implications for the management of neovascular age-related macular degeneration. Ophthalmology.

[6] Dugel PU (2020). HAWK and HARRIER: Phase 3, multicenter, randomized, double-masked trials of brolucizumab for neovascular age-related macular degeneration. Ophthalmology.

[7] Dugel PU (2021). HAWK and HARRIER: Ninety-six-week outcomes from the phase 3 trials of brolucizumab for neovascular age-related macular degeneration. Ophthalmology.

[8] Matsumoto H, Hoshino J, Mukai R, Nakamura K, Akiyama H (2021). Short-term outcomes of intravitreal brolucizumab for treatment-naive neovascular age-related macular degeneration with type 1 choroidal neovascularization including polypoidal choroidal vasculopathy. Sci. Rep..

[9] Ohji M (2020). Efficacy and safety of intravitreal aflibercept treat-and-extend regimens in exudative age-related macular degeneration: 52- and 96-week findings from ALTAIR: A randomized controlled trial. Adv. Ther..

[10] Ohji M (2021). Relationship between retinal fluid and visual acuity in patients with exudative age-related macular degeneration treated with intravitreal aflibercept using a treat-and-extend regimen: Subgroup and post-hoc analyses from the ALTAIR study. Graefes. Arch Clin. Exp. Ophthalmol..

[11] Maruko I, Iida T, Saito M, Nagayama D, Saito K (2007). Clinical characteristics of exudative age-related macular degeneration in Japanese patients. Am. J. Ophthalmol..

[12] Morimoto M, Matsumoto H, Mimura K, Akiyama H (2017). Two-year results of a treat-and-extend regimen with aflibercept for polypoidal choroidal vasculopathy. Graefes. Arch. Clin Exp. Ophthalmol..

[13] Fukuda Y (2021). Comparison of outcomes between 3 monthly brolucizumab and aflibercept injections for polypoidal choroidal vasculopathy. Biomedicines.

[14] Koh A (2012). EVEREST study: Efficacy and safety of verteporfin photodynamic therapy in combination with ranibizumab or alone versus ranibizumab monotherapy in patients with symptomatic macular polypoidal choroidal vasculopathy. Retina.

[15] Koh A (2017). Efficacy and safety of ranibizumab with or without verteporfin photodynamic therapy for polypoidal choroidal vasculopathy: A randomized clinical trial. JAMA Ophthalmol..

[16] Yamamoto A (2015). One-year results of intravitreal aflibercept for polypoidal choroidal vasculopathy. Ophthalmology.

[17] Mones J (2021). Risk of inflammation, retinal vasculitis, and retinal occlusion-related events with brolucizumab: Post hoc review of HAWK and HARRIER. Ophthalmology.

[18] Maruko I (2021). Brolucizumab-related intraocular inflammation in Japanese patients with age-related macular degeneration: A short-term multicenter study. Graefes. Arch. Clin. Exp. Ophthalmol..

[19] Mukai R, Matsumoto H, Akiyama H (2021). Risk factors for emerging intraocular inflammation after intravitreal brolucizumab injection for age-related macular degeneration. PLoS ONE.

[20] Sharma A (2021). Understanding retinal vasculitis associated with brolucizumab: Complex pathophysiology or Occam's Razor?. Ocul. Immunol. Inflamm..

[21] Baumal CR (2021). Expert opinion on management of intraocular inflammation, retinal vasculitis, and vascular occlusion after brolucizumab treatment. Ophthalmol. Retina.

[22] Spaide RF (2020). Consensus nomenclature for reporting neovascular age-related macular degeneration data: Consensus on neovascular age-related macular degeneration nomenclature study group. Ophthalmology.

[23] Iijima H, Iida T, Imai M, Gohdo T, Tsukahara S (2000). Optical coherence tomography of orange-red subretinal lesions in eyes with idiopathic polypoidal choroidal vasculopathy. Am. J. Ophthalmol..

[24] Yanai H (2015). Statcel: The Useful Add-In Software Forms on Excel.

